# The Benefits of an Employee-Friendly Company on Job Attitudes and Health of Employees: Findings from Matched Employer–Employee Data

**DOI:** 10.3390/ijerph19159046

**Published:** 2022-07-25

**Authors:** Raphael M. Herr, Luisa Leonie Brokmeier, Joachim E. Fischer, Daniel Mauss

**Affiliations:** 1 Center for Preventive Medicine and Digital Health (CPD), Medical Faculty Mannheim, Heidelberg University, 68167 Mannheim, Germany; luisa.brokmeier@medma.uni-heidelberg.de (L.L.B.); joachim.fischer@medma.uni-heidelberg.de (J.E.F.); dmousetrap@googlemail.com (D.M.); 2 Department of Medical Informatics, Biometry and Epidemiology, Friedrich-Alexander-Universität Erlangen-Nürnberg (FAU), 91054 Erlangen, Germany

**Keywords:** employee-friendly company, job attitudes, health, matched employer–employee data, human resources (HR) management, company level

## Abstract

Background: This study explored the association of an employee-friendly work environment with employees’ job attitudes (engagement, commitment, turnover intentions, and job satisfaction), and health (mental and general health), applying matched employer–employee data. Methods: The German Linked Personnel Panel (LPP; *n* = 14,182) survey simultaneously captures the data of employees and the human resources (HR) management of companies. A two-step cluster analysis of 16 items of the HR valuation identified relatively more- and less-employee-friendly companies (EFCs). Logistic regressions tested differences between these companies in the assessment of job attitudes and health of their employees. Results: Compared to less-EFCS, more-EFCS had a reduced risk of poorer job attitudes and substandard health of their employees. For example, the risk for higher turnover intentions was reduced by 33% in more-EFCS (OR = 0.683, 95% C.I. = 0.626–0.723), and more-EFCS had an 18% reduced chance of poor mental health reporting of their employees (OR = 0.822, 95% C.I. = 0.758–0.892). Conclusions: More-EFCS have more motivated and healthier employees. The most distinct factors for more-EFCS were: the existence of development plans for employees, opportunities for advancement and development, and personnel development measures.

## 1. Introduction

Job attitudes and the health of employees are central elements for the productivity and success of companies [1]. An adverse work environment represents a considerable risk factor, as it is related to higher levels of ill-health of the employees (e.g., coronary heart disease, musculoskeletal problems, and mental diseases) as well as sickness absence rates and a lower firm productivity [2,3,4,5,6,7]. It is therefore of highest interest of companies to avoid an adverse work environment to prevent health, sickness absence, and related costs to the company [8].

In addition to lower level factors (e.g., individual, group, or leader level), the organizational level can provide further workplace resources, defining the work environment related to both the employee well-being and the organizational performance [9]. Beneficial human resources (HR) management can thus promote organizational performance either directly or through a positive influence on employees’ well-being [10,11]. According to the mutual gains perspective of HR management, HR practices are related to benefits for both employees, in terms of job satisfaction and well-being, and the organization, in terms of productivity [9]. Such employee-friendly companies (EFCs) with supportive HR practices, such as training programs [12] or shared social activities [13], have a better financial performance compared to other companies, which is in line with the “happy worker–productive worker thesis”, and employee-friendliness represents a relevant determinant for positive job attitudes and the health of employees [9,14]. Positive job attitudes, such as employee engagement, in turn, are associated with higher financial turnover, less sickness absence, absenteeism, and presenteeism, and a better operational financial situation [15]. Another study found that employees with higher job satisfaction showed less lost productivity caused by absenteeism or presenteeism [16]. According to Kooij and colleagues, it could be important that companies adapt their HR procedures to different target groups, as the effects might differ [17].

Additional evidence for the positive effects of determinates at the organizational level comes from research on high-performance work practices. According to Ogbonnaya and colleagues (2017) [18], high-performance work practices comprise the following three dimensions of the employees’ working environment: ability (e.g., staff training programs and hiring), motivation (e.g., career development, supportive management), and opportunity (e.g., job autonomy, flexible working). Such high-performance work practices were positively related to employees’ health and well-being [18]. In a longitudinal study, an intervention implementing high-performance work practices was associated with higher productivity three months later [19].

This study aims to extend the research by defining EFCs on a basis of comprehensive HR management information (e.g., development plans, promotion, procedures in case of dissatisfaction, etc.) matched to the employees’ job attitudes (work engagement, commitment, turnover intention, job satisfaction) and health (mental health and general health), and to test their associations. The hypothetical relation of the employee-friendliness of companies with positive job attitudes and better health of employees can guide further research as well as inform about potential practical measures, as the most relevant factors for EFCs will be identified. Considering attitudes and health simultaneously, as in this study, has the advantage of providing information for the design and implementation of interventions that optimize these outcomes simultaneously [10]. By applying a large and Germany-representative employer–employee matched-data dataset, this study contributes to the literature by empirically defining the employee-friendliness of companies and testing the association with work engagement, commitment, turnover intention, job satisfaction, and mental and general health in the German working context.

## 2. Materials and Methods

### 2.1. Study Population

The present study analysed data from the Linked Personnel Panel (LPP) survey. This data links the simultaneous observation of the employer and employee perspective and is considered representative of private German companies with more than 50 employees in the manufacturing and service sectors [20,21,22]. In the current study, employee-level information (i.e., job attitudes and health) were linked with company-level information on HR management practices and structural firm characteristics (i.e., employee friendliness of the company; EFC). This study comprised pooled and linked data, with 14,182 observations from companies and employees which participated in one or more of the three waves (2012/2013, 2014/2015, and 2016/2017). Thus, this study is cross-sectional in nature, and the panel information is not considered here. Participants provided informed consent and the Ethics Committee of the Medical Faculty of the University of Heidelberg approved the secondary data analysis (2018-514N-MA).

### 2.2. Measurement

**Employee-friendliness of the company (EFC).** The following items from the company questionnaire answered by the HR management of the companies were used to estimate the employee-friendliness of the companies (EFC):

- Development plans for employees exists (yes vs. no)
- Employee surveys were conducted (yes vs. no)
- Promotion of further training to higher educational qualifications (yes vs. no)
- If dissatisfied with performance: discussion with employees (1 = does not apply to 5 = fully applies)
- If dissatisfied with performance: personnel development measures (1 = does not apply to 5 = fully applies)
- If dissatisfied with performance: different position in the company (1 = does not apply to 5 = fully applies)
- How important when promoting employees: personal competence (1 = important to 5 = irrelevant)
- Importance in employee promotion: ethical values competence (1 = important to 5 = irrelevant)
- Importance for employee loyalty: remuneration (1 = unimportant to 5 = very important)
- Importance for employee loyalty: flexible working hours (1 = unimportant to 5 = very important)
- Importance for employee loyalty: additional benefits (1 = unimportant to 5 = very important)
- Importance for employee loyalty: opportunities for advancement and development (1 = unimportant to 5 = very important)
- Importance for employee loyalty: working atmosphere (1 = unimportant to 5 = very important)
- Importance for employee loyalty: compatibility of family and work (1 = unimportant to 5 = very important)
- Importance when filling a position: personal competence (1 = important to 4 = irrelevant)
- Importance when filling a position: ethical values (1 = important to 4 = irrelevant)

**Mental health.** Mental health was measured by the WHO-5-Well-Being Questionnaire [23]. Participants rated on a six-point Likert-scale (0 = “at no time” to 5 = “all of the time”) five statements about how they had felt over the preceding two weeks: cheerful and in good spirits; calm and relaxed; active and vigorous; woke up feeling fresh and rested; and daily life has been filled with things that interest me (Cronbach’s alpha = 0.84). An index ranging from 0–100 was computed by summation of the items multiplied by four, and the established cut-off of ≥51.0 was applied to define better mental health [23].

**General health.** Employees rated their general health by answering the question: “How would you describe your current state of health?” on a five-point Likert-scale. “Very good health” and “good health” were categorised as “good health”, while “bad”, “less well”, and “satisfactory health” were categorised as “poor health”.

**Work engagement.** Work engagement was measured by the Utrecht Work Engagement Short Scale (UWES-9) with the dimensions of dedication and absorption [24]. The employees rated nine statements according to how often they felt that way about their work (e.g., “at my work, I feel bursting with energy”) on a five-point Likert-scale (1 = “every day”, 2 = “a few times per week”, 3 = “a few times per month”, 4 = “a few times per year”, 5 = “never”). The items were reversed, and a mean score was calculated (Cronbach’s alpha = 0.91) and categorised in high and low engagement by median split.

**Commitment.** Commitment was measured by the six item version of Meyer et al. [25] on a five-point Likert scale (Cronbach’s alpha = 0.83). A mean score was calculated and split by median to define high vs. low commitment.

**Turnover intention.** Turnover intention was measured by the question: “How many times in the past 12 months have you thought about changing your job?” [26]. Answers were rated on a 5-point Likert scale ranging from 1 (“daily”) to 5 (“never”). A median spilt defined low and high turnover intentions.

**Job satisfaction.** Job satisfaction was assessed by the item: “How satisfied are you today with your job?”, measured on an 11-point Likert scale ranging from 0 (“completely unhappy”) to 10 (“completely happy”) [27]. High vs. low job satisfaction was defined by median split.

### 2.3. Statistical Analyses

Cluster analyses were applied to define EFCs. The 16 items relevant for employee-friendliness of companies were z-transformed, and a two-step clustering approach was used by combining Ward’s hierarchical clustering with non-hierarchical K-means clustering [28,29]. To avoid potential suboptimal solutions of hierarchical models, the results from the Ward’s hierarchical clustering were used as the cluster centres for non-hierarchical k-means clustering. The number of clusters was determined by the Calinski/Harabasz pseudo-F to indicate most distinct clustering.

The effect size (Cohen’s d) was calculated to determine how distinct the individual items between groups were. From the items with the highest distinctiveness (i.e., strong Cohen’s d of >0.80), a sum score was calculated and split into quartiles for further analyses.

Associations of the groups with health and job attitudes were estimated by logistic regression models. The starting model (Model 0) was unadjusted, while Model 1 controlled for age, gender, white- or blue-collar occupation, company size, and industry, to take into account potential group differences [17].

## 3. Results

### 3.1. Clustering EFCs

**Figure 1:**
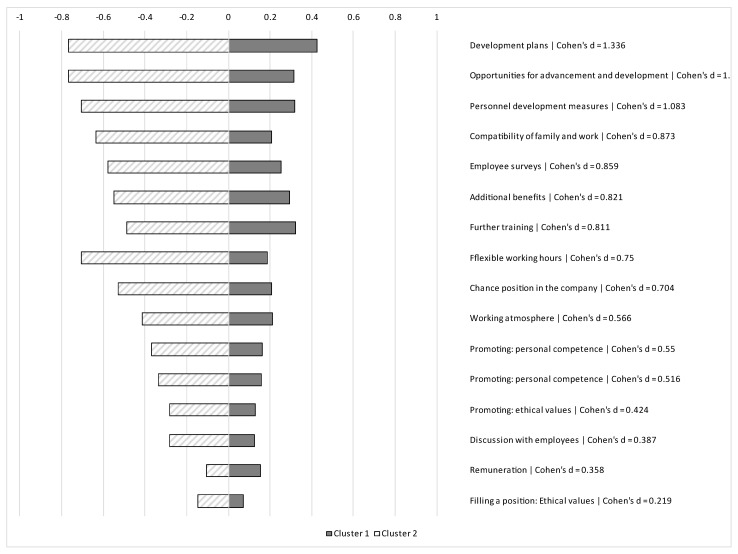
Results of the cluster analysis based on the HR management survey. Mean values of the z-scores for both clusters; Cluster 1: *n* = 7378, Cluster 2: *n* = 3569.

The two-cluster solution yielded the most distinct solution (highest Calinski/Harabasz pseudo-F). The mean values for both clusters and the effect sizes for the individual items are shown in Figure 1. The most distinctive factors (Cohen’s d > 0.80) were: development plans, opportunities for advancement and development, personnel development measures, compatibility of family and work, employee surveys, additional benefits, and further training. Cluster 1 was interpreted as more-EFCS, while cluster 2 was interpreted as less-EFCS.

### 3.2. Description of the Clustered EFCs

The description of the two clusters is presented in Table 1. More-EFCS had, on average, slightly younger and more male employees in a rather white-collar occupation. Most of the more-EFCS had more than 500 employees (46%), while most of the less-EFCS had less than 250 employees (63%). Both types were mostly in the manufacturing industry; however, the percentage was higher in more-EFCS.

### 3.3. Associations of EFCs with Health and Job Attitudes of Employees

Compared to less-EFCS, more-EFCS had an 18% reduced chance (OR = 0.822, 95% C.I. = 0.758–0.892) that their employees reported poor mental health (Table 2, Model 0). For poor self-rated health, the risk was reduced by 15% (OR = 0.849, 95% C.I. = 0.791–0.912). Furthermore, in more-EFCS, the chance that employees showed increased turnover intention was reduced by 33%, low commitment by 29%, and low job satisfaction by 25%. The chance of low engagement was reduced by 7%. These associations were largely independent of the age, sex, or type of occupation (white- vs. blue-collar) of the employee as well as the company size and industry (Table 2, Model 1 adjusted analyses). However, the relations were somewhat attenuated, and self-rated health did not reach the p < 0.05 level of significance.

### 3.4. Associations of the Quartiles of the Most Distinctive Factors for EFCs with Employee’s Health and Job Attitudes

The results of the analyses of the quartiles of the sum score of the seven most distinctive items (Cohen’s d > 0.80) are displayed in Table 3. Overall, it was shown that the higher the quartile (i.e., the more employee-friendly the company is) the lower the risk was for bad health and worse job attitudes. Furthermore, a dose–response relationship was observed. For example, the risk for poor mental health was reduced by 19% in the highest quartile compared to the lowest (Model 0). The reduction in the third quartile was 18%, and in the second it was 14%. For poor self-rated health, the risk reduction was 26% in the highest quartile compared to the lowest. The reduced risk for low commitment was 31%, for high turnover intention it was 33%, and for low job satisfaction it was 22% in the highest quartile compared to the lowest. Only for engagement, no significant reduction was found in the unadjusted model. However, after adjustment for age, sex, white- or blue-collar occupation, company size, and industry (Model 1), the risk of low engagement was reduced by 11%. For the other outcomes, the extent of the risk reduction was comparable after adjustment (Model 1).

## 4. Discussion

This study has shown that more employee-friendly companies (EFCs) have healthier and more motivated and engaged employees. We thus can provide additional support for the “mutual gains perspective”, as the employee-friendliness of companies has been confirmed here as a relevant determinant for positive job attitudes and the health of employees [9,14].

The most pronounced differences in the examined outcomes between more and less-EFCS were found in a reduced risk for higher turn-over intention, lower commitment, and lower job satisfaction, followed by lower mental and general health and engagement. We cannot provide a clear explanation of why the associations with the outcomes differed. While self-rate health represents a very rough measurement, which was only assessed by a single item and might be influenced by many more factors than the employee-friendliness of the company, we can only speculate as to why the association of EFCs with engagement was relatively small. The associations became somewhat stronger when analyses were adjusted for age, sex, white- or blue-collar occupation, company size, and industry, and it might be assumed that some of these factors might serve as moderators of the association.

We identified the following variables as most important for EFCs: development plans for employees, opportunities for advancement and development, personnel development measures, compatibility of family and work, employee surveys, additional benefits, and further training to advance educational qualifications. These seven factors appeared sufficient to define more-EFCS, and as more of these factors were implemented by companies, their employees were healthier and more motivated and committed (i.e., a dose-response relationship occurred). The associations were largely independent from industries, size of company, and age and sex of employees. In consequence, companies should be motivated to use (at least) some of these seven ways to increase their employee-friendliness and gain healthy and productive employees.

In line with previous research, this study provided further evidence for the relevance of organizational-level factors for job attitudes and health [9]. However, potential combined effects with lower-level factors, for example, at the individual, group, or leader level, could not be tested here, and further studies are needed to examine potential interactive effects of workplace resources at the different levels.

The job attitudes studied here were all associated with the employee-friendliness of companies, and all show a relation to productivity. An engaged employee, for example, knows the business context, and tries to improve his performance, which has beneficial effects for the company and a direct impact on the productivity [30]. Engagement might increase productivity through multiple pathways. Engagement is related to customer satisfaction, which is relevant for the success of a company, and employees’ engagement is also linked to improved job commitment and involvement, creating a motivated workforce that tries to achieve the company’s goals and to promote its success [30]. EFCs might thus create a work environment which increases the intellectual commitment as well as emotional attachment of the employees to the company. One reason for this might be the increased opportunities for participation and involvement of employees in EFCs, as these factors are suggested to have a positive effect on employees’ job satisfaction, commitment, and productivity [31,32,33]. Another job attitude linked to the employee-friendliness of a company, turnover intentions, represents another critical factor for the productivity of companies, as employees’ intention to leave represents a very costly issue and has a profound influence on the companies’ performance and productivity [34]. Potential moderating factors for this association are higher job stress and lower job satisfaction [34,35]. Higher job satisfaction also has more direct effects on productivity, such as by reduced productivity-related costs and presenteeism costs [36]. Taken together, the measured job attitudes in this study appear to have direct or indirect associations with the performance and productivity of a company; however, additional research might be beneficial to develop and establish a conceptual framework for the direction and order of the effects.

More-EFCS differed form less-EFCS according to some descriptive characteristics. In more-EFCS, employees were slightly younger, more likely to be male, and more likely to be in a white-collar occupation. Furthermore, more-EFCS were bigger companies with more employees than less-EFCS, while less-EFCS were more active in the service sector. As no typification of the different EFCs appears possible on the basis of this description, future studies should try to consider more factors that could allow a more precise identification of EFCs.

While a general consensus exists in the literature that HR management and HR development is related to productivity and well-being of employees (e.g., [9,10,37,38]), we were able to expand previous knowledge with this study by taking advantage of a representative and matched employer–employee data set with a detailed survey of HR practices and job attitudes and health of employees. Thus, it was possible to simultaneously test the associations of the employee-friendliness with health and job attitudes, showing that there was a positive relation to both kinds of outcomes at the same time. Furthermore, we were able to confirm and extend the list of related outcomes (e.g., mental health) and to transfer previous findings to the German work context in a representative sample. A large study from the United Kingdom found high-performance work practices to be related to employee’s health and well-being [18]. In our German sample, there was also a robust association of the employee-friendliness of companies with general health, but also with mental health. The results thus appear not to be country-specific, and the concern that there might be a possibility of negative effects on employee health of HR management was not supported in this study [10]. However, further studies appear necessary to test the relation in different countries and cultural settings, and, up to now, the literature has been dominated by a diverse range of different expressions and concepts, which essentially describe a similar construct (e.g., high-performance work practices, HR management, EFCs). Therefore, efforts should be made to develop and define a uniform construct and designation.

This study provides information about potential practical implications. Companies should be motivated to enhance their employee-friendliness, as it has positive effects for their employees in terms of better health and positive job attitudes. Furthermore, as outlined above, more positive job attitudes such as work engagement and job satisfaction are associated with higher productivity and greater financial turnover for the company [15,19], and interventions focused on developing personal resources and capabilities as well as leadership training and organizational-level learning have proven to be effective for employees’ well-being [37,38]. The management might thus use the list of factors identified in this study as most important for EFCs and might try to implement the most relevant points if they are not already present in their company. For example, realizing development plans for employees or opportunities for advancement and development seems to be easily implementable and appears to have far-reaching and positive consequences.

This study has some limitations. The first limitation refers to the cross-sectional analyses of the study, which prohibits drawing causal conclusions. However, as the findings are based on matched employer–employee data, the risk of a common method bias seems reduced [39]. Another limitation refers to the selection of the factors exploitable for the clustering of the companies in more or less-EFCS. This selection was redistricted by the questions asked of the HR management in the LPP survey. Thus, other possibly relevant aspects could not be considered in this study, and there might be other very relevant factors. Future studies should examine this.

## 5. Conclusions

The results of this study highlight the great benefit for companies to be employee-friendly. Based on matched employer–employee data, we found that more-EFCs have healthier employees with a higher commitment, job satisfaction, and engagement, and lower turnover intentions.

## Figures and Tables

**Table 1:** Description of the study population according to the clusters.

		Cluster 1: More-EFCS		Cluster 2: Less-EFCS		Test Value	*p*-Value
Age (years, mean, SD)		46.54	10.35	47.10	10.25	−3.07	0.0022
Sex (male, %, n)		73.83	7066	69.99	3232	23.18	<0.001
Type of occupation(white-collar, %, n)		63.57	6083	54.28	2506	112.57	<0.001
Company size (%, n)						1900.00	<0.001
	<250	26.19	2506	62.73	2897		
	250–499	27.45	2627	20.51	947		
	≥500	46.36	4437	16.76	774		
Industry (%, n)						69.02	<0.001
	Manufacturing industry	71.62	6854	66.02	3049		
	Trade, transport, news	9.66	924	13.97	645		
	Business-related services	18.73	1792	20.01	924		

Test value: *t*-test for continuous variables, Chi^2^ for categorical variables.

**Table 2:** Associations of EFCs (more vs. less) with health and job attitudes of employees.

	Model 0				Model 1			
	OR	95% Conf. Interval		*p*-Value	OR	95% Conf. Interval		*p*-Value
Poor mental health	0.822	0.758	0.892	<0.001	0.872	0.798	0.952	0.002
Poor self-rated health	0.849	0.791	0.912	<0.001	0.941	0.870	1,019	0.135
Low engagement	0.925	0.861	0.993	0.031	0.866	0.801	0.936	<0.001
Low commitment	0.706	0.658	0.758	<0.001	0.754	0.697	0.816	<0.001
High turnover intention	0.673	0.626	0.723	<0.001	0.725	0.668	0.786	<0.001
Low job satisfaction	0.753	0.701	0.809	<0.001	0.800	0.740	0.865	<0.001

OR = odds ratio. Model 0 = unadjusted. Model 1 = adjusted for age, gender, white- or blue-collar, company size, industry.

**Table 3:** Associations of the quartiles of the seven most distinctive factors for ECFs with employees’ health and job attitudes.

	Model 0				Model 1			
	OR	95% Conf. Interval		*p*-Value	OR	95% Conf. Interval		*p*-Value
Mental health								
(1) Lowest	1				1			
(2)	0.865	0.785	0.953	0.003	0.901	0.815	0.996	0.041
(3)	0.821	0.745	0.905	<0.001	0.864	0.780	0.957	0.005
(4) Highest	0.815	0.727	0.914	<0.001	0.856	0.760	0.964	0.011
Self-rated health								
(1) Lowest	1							
(2)	0.843	0.774	0.918	<0.001	0.910	0.832	0.995	0.039
(3)	0.886	0.814	0.964	0.005	0.973	0.889	1.066	0.559
(4) Highest	0.736	0.666	0.814	<0.001	0.820	0.737	0.912	<0.001
Engagement								
(1) Lowest	1							
(2)	0.930	0.854	1.012	0.094	0.884	0.810	0.965	0.006
(3)	0.929	0.854	1.011	0.088	0.882	0.807	0.964	0.006
(4) Highest	0.947	0.858	1.045	0.276	0.890	0.803	0.986	0.026
Commitment								
(1) Lowest	1							
(2)	0.772	0.709	0.840	<0.001	0.793	0.725	0.867	<0.001
(3)	0.677	0.623	0.737	<0.001	0.718	0.656	0.786	<0.001
(4) Highest	0.688	0.623	0.758	<0.001	0.713	0.642	0.791	<0.001
Turnover intention								
(1) Highest	1							
(2)	0.794	0.729	0.865	<0.001	0.830	0.757	0.910	<0.001
(3)	0.670	0.615	0.731	<0.001	0.724	0.659	0.795	<0.001
(4) Lowest	0.675	0.610	0.746	<0.001	0.689	0.617	0.768	<0.001
Job satisfaction								
(1) Lowest	1							
(2)	0.823	0.755	0.898	<0.001	0.858	0.785	0.938	0.001
(3)	0.760	0.697	0.829	<0.001	0.806	0.736	0.882	<0.001
(4) Highest	0.780	0.706	0.863	<0.001	0.819	0.738	0.909	<0.001

OR = odds ratio. Model 0 = unadjusted. Model 1 = adjusted for age, gender, white- or blue-collar, company size, industry.

## Data Availability

The data used in this study are available from the Research Data Center (FDZ) of the German Federal Employment Agency (BA) at the Institute for Employment Research (IAB). Data access can be requested from the Research Data Center (FDZ) of the German Federal Employment Agency (BA) at the Institute for Employment Research (IAB): https://fdz.iab.de/.
